# Predicting Visual Field Loss in Glaucoma Using OCT and Deep Learning: A Comparative Study of U-Net Variants

**DOI:** 10.1016/j.xops.2026.101169

**Published:** 2026-03-23

**Authors:** Kyoung Ohn, Jiwook Hwang, Jiwon Jung, Woong-Joo Whang

**Affiliations:** 1 Department of Ophthalmology, Yeouido St. Mary’s Eye Hospital, College of Medicine, The Catholic University of Korea, Seoul, Republic of Korea; 2 PuzzleAI Co., Ltd., Seoul, Republic of Korea

**Keywords:** Glaucoma, OCT, Visual field prediction, Deep learning, Generative models

## Abstract

**Purpose:**

Glaucoma is a chronic eye disease that progressively damages the optic nerve, leading to irreversible visual field (VF) loss. OCT and VF tests are essential for monitoring structural and functional changes in glaucoma. This study applies 3 deep learning models—R2 U-Net, Dense U-Net, and Nested U-Net (UNet++)—to predict VF outcomes using retinal nerve fiber layer (RNFL) thickness maps from OCT images.

**Design:**

A retrospective cross-sectional study.

**Subjects:**

A total of 1640 patients with glaucoma diagnosed at a tertiary referral center were included. Only 1 eye (left eye) per patient was analyzed to avoid intereye correlation. Eyes included patients with early, moderate, and advanced glaucoma.

**Methods:**

We used a dataset of OCT and VF data from 1640 glaucoma patients, divided into training, validation, and test sets. The 3 deep learning models were trained and evaluated using 5 performance metrics: mean squared error (MSE), mean absolute error (MAE), peak signal-to-noise ratio (PSNR), structural similarity index (SSIM), and Fréchet inception distance (FID). The goal was to predict VF outcomes based on OCT-derived RNFL thickness maps.

**Main Outcome Measures:**

Accuracy and image quality of predicted VF maps compared with ground truth VF maps, assessed by MSE, MAE, PSNR, SSIM, and FID.

**Results:**

R2 U-Net outperformed all other architectures, with the lowest MSE and MAE values and the highest SSIM and PSNR scores, indicating superior accuracy and image quality. Nested U-Net and Dense U-Net lagged, with Dense U-Net showing the lowest predictive accuracy.

**Conclusions:**

This study is the first to apply generative artificial intelligence (AI) models, such as R2 U-Net, to predict VF loss based on OCT data, with both models demonstrating exceptional performance. These findings highlight the potential of generative AI to enhance glaucoma diagnosis and facilitate personalized treatment planning.

**Financial Disclosure(s):**

The authors have no proprietary or commercial interest in any materials discussed in this article.

Glaucoma is a chronic eye disease that causes damage to the optic nerve, leading to visual field (VF) loss, and is one of the leading causes of blindness worldwide.^1^ Visual field loss initially manifests subtly, and if not diagnosed early, it can progressively worsen, resulting in irreversible vision impairment.^2^ For this reason, early diagnosis and continuous monitoring of glaucoma are essential. Two key tools in diagnosing and monitoring the progression of glaucoma are the visual field test and OCT, which assess visual function loss and structural changes in the optic nerve, respectively.^3^^,^^4^

Understanding the structure–function relationship between VF testing and OCT is crucial for diagnosing and developing treatment plans for glaucoma. The VF test assesses visual function loss resulting from optic nerve damage, while OCT measures the thickness of the optic nerve head and retinal nerve fiber layer (RNFL) to detect structural changes in the optic nerve. By analyzing the relationship between these 2 measurement methods, a more precise link between VF loss and structural damage to the optic nerve can be established. The study by Garway-Heath et al^5^ provided a foundational mapping of the anatomical relationship between VF points and the optic nerve head, offering an important basis for understanding the structure–function relationship.

Deep learning technology has made significant advancements in the field of medical image analysis, particularly in learning complex relationships between data, surpassing traditional statistical models. Deep learning has become a powerful tool for enhancing predictive performance by learning patterns from medical data. This technology is being used to predict VF loss based on OCT and to better understand the relationship between optic nerve damage and functional visual impairment. Models like U-Net have been particularly successful in medical image processing. U-Net utilizes an encoder–decoder structure to learn complex image features and is employed to predict visual function loss. Variations of U-Net, such as Dense U-Net, Nested U-Net, R2 U-Net, and Res U-Net are designed to efficiently learn and predict critical features in medical data through deep network structures, skip connections, recurrent feedback, and gating mechanisms.

The application of deep learning in glaucoma research has also demonstrated significant advances, particularly in predicting VF outcomes from OCT data. For instance, Kamalipour et al^6^ developed a deep learning model that uses OCT data to predict VF results and estimated central VF maps based on RNFL thickness measurements, outperforming traditional methods in accuracy and improving predictions of visual function. Similarly, Christopher et al^7^ applied deep learning approaches to OCT optic nerve head images and RNFL thickness maps, achieving high accuracy in predicting glaucomatous VF damage and estimating severity. These studies collectively highlight the growing utility of deep learning in automating and enhancing glaucoma diagnosis and monitoring, offering new tools for clinicians to individualize patient care and streamline diagnostic processes.

Recent advances in artificial intelligence (AI) have led to increasing interest in predicting VF outcomes from OCT-derived structural information. Several prior studies have demonstrated the feasibility of AI-based approaches for glaucoma detection and functional assessment. Hood et al^8^ proposed an OCT-based framework for glaucoma detection using RNFL probability maps, highlighting the diagnostic utility of deep learning models in distinguishing glaucomatous damage from normal anatomical variation. However, their work primarily focused on disease detection and classification rather than direct prediction of VF outcomes. More recently, Chuangsuwanich et al^9^ introduced a geometric deep learning approach using Point-Net to predict VF pattern standard deviation (PSD) maps by integrating biomechanical features, such as intraocular pressure (IOP)–induced neural tissue strain, with structural optic nerve head information. Although this approach demonstrated improved predictive performance compared with structure-only models, it relied on specialized biomechanical measurements obtained through acute IOP modulation, which may limit its applicability in routine clinical practice.

Despite these advances, limitations remain in existing approaches for VF prediction. Traditional statistical methods or simple structure–function mapping techniques often fail to adequately account for individual anatomical variability, which can hinder predictive accuracy. Moreover, although recent AI-based studies have demonstrated the feasibility of predicting VF outcomes from OCT data, relatively few have focused on directly generating complete VF pattern images using routinely acquired OCT measurements alone. In particular, generative image-to-image deep learning approaches and systematic comparisons of architectures optimized for medical image translation tasks remain underexplored.

Therefore, a new approach is needed to improve diagnostic accuracy. This study aims to develop a more accurate VF prediction model by mapping OCT-based RNFL thickness maps to VF tests using 3 deep learning models: Dense U-Net, Nested U-Net, and R2 U-Net. The study proposes personalized VF prediction model based on each patient's structural data, and the performance of each model will be compared using various evaluation metrics.

## Methods

### Subjects

Among 1640 patients diagnosed with glaucoma at Yeouido St. Mary's Hospital between March 2019 and December 2024, were included. Glaucoma diagnosis included primary open-angle glaucoma, normal-tension glaucoma, or primary angle-closure glaucoma. Subjects consisted of 965 eyes of those with normal-tension glaucoma, 634 eyes of those with primary open-angle glaucoma, and 44 eyes of those with primary angle-closure glaucoma.

Glaucoma was defined by glaucomatous-appearing VF defects with the presence of a compatible glaucomatous optic disc that showed diffuse or localized rim thinning, a notch in the rim, a vertical cup-to-disc ratio ≥0.7 or ≥0.2 compared with the other eye or RNFL defect. Glaucomatous-appearing VF defects were confirmed by at least 2 reliable Swedish Interactive Threshold Algorithm 24-2 examinations (defined as a false-negative rate of <15%, a false-positive rate of <15%, and fixation losses of <20%) and defined if they met 2 of the following 3 criteria: (1) a cluster of 3 points with a probability of lower than 5% on the pattern deviation map in at least 1 hemifield and including at least 1 point with a probability of lower than 1% or a cluster of 2 points with a probability of lower than 1; (2) glaucoma hemifield test results outside normal limits; and (3) a PSD outside 95% of the normal limits.

All diagnoses were made by a single glaucoma specialist (K.O.) using consistent diagnostic criteria. Primary open-angle glaucoma was defined as glaucoma with a baseline IOP >21 mmHg prior to treatment, an open anterior chamber angle on gonioscopy, and no secondary cause of optic nerve damage. Normal-tension glaucoma was diagnosed in eyes with untreated IOP ≤21 mmHg, an open angle on gonioscopy, and glaucomatous optic disc and VF changes without other identifiable causes of optic neuropathy. Primary angle-closure glaucoma was defined as glaucoma with appositional contact between the peripheral iris and the trabecular meshwork over more than 270° on gonioscopy, accompanied by glaucomatous optic disc damage and corresponding VF defects.

Using the Hodapp–Parrish classification, glaucomatous eyes were stratified into 3 groups based on VF mean deviation (MD): early glaucoma (MD > –6 decibels [dB]), moderate glaucoma (–12 dB < MD ≤ –6 dB), and severe glaucoma (MD ≤ –12 dB).^10^

The following exclusion criteria were applied to minimize influence of confounding factors: (1) patients with history of any retinal disease, including the diabetic retinopathy and myopic macular degeneration, (2) patients with history of eye trauma or surgery, excluding the uncomplicated cataract surgery, (3) patients with any optic nerve disease, except for the glaucoma itself, (4) patients with history of systemic or neurologic diseases, which might have been related to the VF defect.

We retrospectively enrolled all consecutive eligible patients willing to participate, all of whom provided written consent. This retrospective study was approved by the Institutional Review Board of Yeouido St. Mary’s Hospital and was conducted in accordance with the tenets of the Declaration of Helsinki (IRB No. SC24RISI0114). On the same day, VF testing was conducted using the Swedish Interactive Threshold Algorithm Standard 24-2 on a Humphrey Field Analyzer (Carl Zeiss Meditec), and retinal nerve fiber layer (RNFL) imaging was performed using OCT with a Cirrus OCT device (Carl Zeiss Meditec).

### Data Collection and Dataset Construction

OCT scans and Humphrey VF test data from 1640 glaucoma patients were included in this study. To avoid potential bias due to intereye correlation, only 1 eye (left eye) per patient was included in the analysis. As a result, each data point represented a unique patient, and no subject contributed more than 1 sample to the dataset. Repeated measurements were not included in the analysis; for each patient, only a single OCT–VF pair obtained at 1 time point was used.

The dataset was divided into training (70%), validation (20%), and test (10%) sets using a predetermined ratio applied consistently across all experiments. Data splitting was performed at the patient level to prevent data leakage. The training set was used for model training, the validation set for hyperparameter tuning and model selection, and the test set was strictly held out for performance evaluation.

### OCT

OCT imaging was performed using the Cirrus HD-OCT system (Carl Zeiss Meditec). All scans were acquired using the Optic Disc Cube 200 × 200 protocol, which covers a 6 × 6 mm field of view centered on the optic nerve head. This protocol consists of 200 A-scans × 200 B-scans, providing volumetric sampling of the peripapillary region. From these scans, RNFL thickness deviation maps were generated. These maps are color-coded probability maps derived by comparison with the device's built-in normative database. Only well-focused, well-centered, high-signal strength (signal strength ≥7) images without eye movement artifacts and segmentation errors were used in analyses.

### Perimetry

Standard automated perimetry was performed using the Humphrey Field Analyzer (Carl Zeiss Meditec) with the Swedish Interactive Threshold Algorithm Standard 24-2 testing strategy. The 24-2 protocol evaluates 54 test locations within the central 24° of the VF, excluding the blind spot. Visual field outputs were represented as grayscale maps derived from threshold sensitivity values, providing a spatial representation of functional visual sensitivity.

### Image Preprocessing for Deep Learning

The RNFL deviation maps were exported at an original resolution of approximately 400 × 400 pixels, while the VF grayscale maps had an original resolution of approximately 300 × 300 pixels. For deep learning analysis, all images were resized to a unified resolution of 256 × 256 pixels using bilinear interpolation to ensure consistency across input–output pairs. Pixel intensity values were subsequently rescaled to the [0, 1] range prior to model training.

### Model Architecture

In this study, 3 deep learning–based models were applied to predict a 1:1 correspondence between OCT-derived RNFL thickness maps and visual sensitivity map. Each model was designed to take OCT data as input and predict VF images, and their performances were compared to identify the optimal prediction model.

Three deep learning models were employed to predict VF test results: R2 U-Net, Dense U-Net, and Nested U-Net (UNet++). These models were selected due to their proven efficacy in medical image processing tasks, particularly in extracting detailed structural features from complex datasets. Each model is based on the U-Net architecture, which utilizes an encoder–decoder structure to process OCT images and generate VF test predictions. The encoder compresses the input image to capture essential features, while the decoder reconstructs the image, focusing on restoring the key visual elements needed for accurate predictions.

(1) Dense U-Net

Dense U-Net combines the DenseNet and U-Net architectures to maximize information flow between layers.^11^^,^^12^ Like U-Net, it follows an encoder–decoder structure, but incorporates DenseNet's key idea, where each layer references all previous layers. This reduces information loss and allows the network to learn more efficiently. By referencing all previous layers, dense connections preserve fine features of the OCT image, leading to more accurate VF predictions.

(2) Nested U-Net (UNet++)

Nested U-Net (UNet++) is an expanded version of U-Net that utilizes multiple skip connections across different layers to minimize information loss.^13^ This structure is highly advantageous in learning and restoring complex image features, as it effectively combines features from different resolutions.

(3) R2 U-Net (Recurrent Residual U-Net)

R2 U-Net is an extension of the U-Net model that uses recurrent residual connections.^14^ This model repeatedly applies the same structure, enabling it to learn deeper and more complex feature representations. Recurrent connections allow the network to capture more intricate patterns within the OCT images, while residual connections preserve crucial visual features, ensuring that the model retains important information across multiple layers.

### Performance Evaluation Metrics

In this study, the predictive performance of the models was evaluated using 5 quantitative metrics. These metrics measure the differences and similarities between the generated VF test images and the actual VF test images from various aspects.

(1) Mean Squared Error

Mean squared error (MSE) is a metric used to evaluate the error of the predictive model by calculating the average of the squared differences between the predicted values and the actual values.^15^ Mean squared error is useful in measuring the overall size of the prediction error when evaluating model performance. In image generation tasks, particularly in medical imaging, an MSE value between 0.001 and 0.01 is generally considered satisfactory, meaning that the model performs reasonably well, but some noticeable errors may still exist. When the MSE drops below 0.001, this is considered excellent performance.

$$\text{MSE}=\frac{1}{N}\;\sum_{i=1}^{n}{\left(xi–yi\right)}^{2}$$

xi, yi: Pixel values of the original and predicted images.

N: Total Number of Pixels

(2) Mean Absolute Error

Mean absolute error (MAE) measures the average of the absolute differences between the predicted values and the actual values, providing a straightforward expression of the model's error.^16^ This metric calculates how far the predicted values deviate from the actual values on average, and the smaller the value, the more accurate the predictions. In the context of image generation, an MAE value between 0.01 and 0.05 is regarded as satisfactory. When the MAE falls below 0.01, it is considered excellent.

$$\text{MAE}=\frac{1}{N}\;\sum_{i=1}^{n}|xi–yi|$$

xi, yi: Pixel values of the original and predicted images.

N: Total Number of Pixels.

(3) Structural Similarity Index

Structural similarity index (SSIM) is a metric that measures the structural similarity between 2 images.^17^ Structural similarity index is designed to mimic the way the human visual system evaluates image quality by considering not only pixel-based differences but also structural information such as luminance, contrast, and texture. Structural similarity index is primarily used to assess the quality of image restoration, reconstruction, or generation. Structural similarity index values range from 0 to 1, with higher values indicating greater similarity between the 2 images. Structural similarity index values between 0.8 and 0.9 are typically deemed satisfactory, meaning that the generated images are reasonably similar to the actual images. When SSIM values exceed 0.9, this is considered excellent.

$$\text{SSIM}=\left(x,y\right)=\left(2\mu_{x}\mu_{y}+C_{1}\right)\left(2\sigma_{\text{xy}}+C_{2}\right)/\;\left(\mu_{x}^{2}+\mu_{y}^{2}+C_{1}\right)\left(\sigma_{x}^{2}+\sigma_{y}^{2}+C_{2}\right)$$

μ_x_, μ_y_: Mean luminance of images x and y.

σx^2^, σ_y_^2^: Variance (contrast) of images x and y.

σ_xy_: Covariance (structural similarity) between images x and y.

C1, C2: Small constants to avoid division by zero (typically used to stabilize the division).

(4) Peak Signal-to-Noise Ratio

Peak signal-to-noise ratio (PSNR) is a metric used to measure the difference between 2 images, often used in evaluating the quality of restored, compressed, or generated images.^18^ Peak signal-to-noise ratio evaluates how similar the reconstructed image is to the original, with higher values indicating better quality. Peak signal-to-noise ratio is measured in dB, with higher values signifying smaller differences between the original and predicted images. If the PSNR exceeds 30 dB, this is considered excellent, showing that the generated image is of very high quality, almost indistinguishable from the original.

$$\text{PSNR}=10\cdot \log\;10\;\frac{{\mathrm{MAX}}^{2}}{MSE}$$

MAX: The maximum possible pixel value in the image (e.g., 255 for an 8-bit image)

MSE: Mean squared error between the 2 images

xi, yi: Pixel values of the original and predicted images.

N: Total number of pixels.

(5) Fréchet Inception Distance

Fréchet inception distance (FID) measures the difference in distribution between 2 sets of images, typically used to assess the quality of generated images relative to real images.^19^ Fréchet inception distance computes the difference between the mean and covariance of 2 distributions in an embedding space, where smaller values indicate that the generated images are more similar to the real ones. An FID score below 10 is considered excellent, meaning that the generated images are nearly indistinguishable from real images in terms of distribution.

FID=∣∣μr–μg∣∣2+Tr(Σr+Σg–2(ΣrΣg)1/2)

μ_r_, μ_g_: Mean vectors of the real and generated images in the embedding space.

Σ_r_, Σ_g_: Covariance matrices of the real and generated images.

T_r_: Trace of a matrix (sum of diagonal elements)

### Analysis of Optimal Epoch and Predicted Image Quality

**Figure 1:**
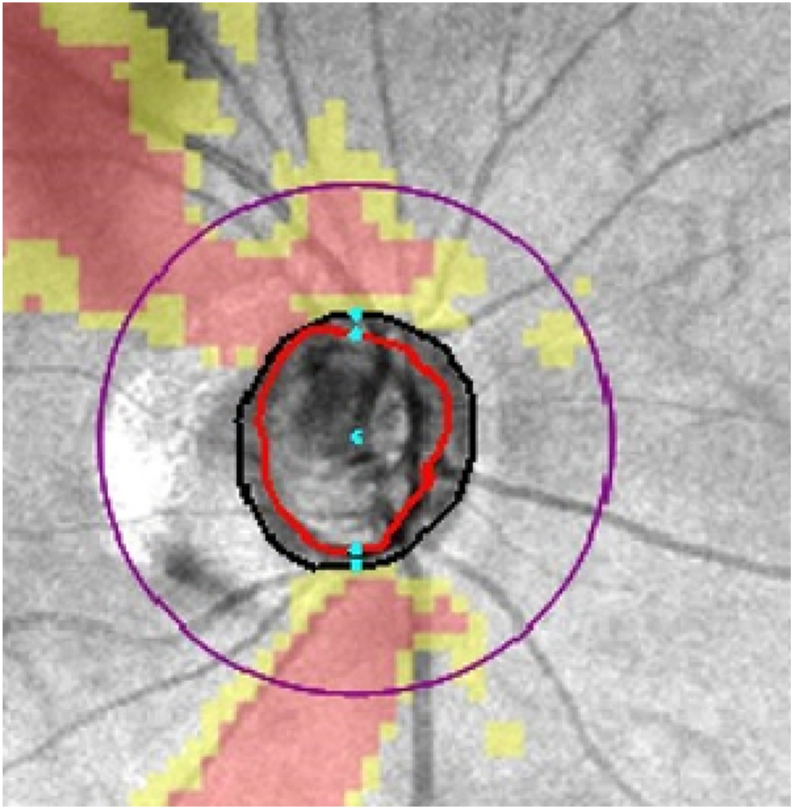
Retinal nerve fiber layer OCT image used as an example to generate predictions across the 3 architectures: R2 U-Net, Dense U-Net, and Nested U-Net.

The upper limit for epochs was set to 200, and every 25 epochs, RNFL OCT image (Fig 1) was used as example to generate predictions across the 3 architectures: R2 U-Net, Dense U-Net, and Nested U-Net. These predicted images were compared to observe changes in quality as the training progressed. Additionally, the changes in the 5 performance metrics (MSE, MAE, SSIM, PSNR, and FID) were monitored on the test set at every 25 epochs. The epoch with the smallest validation loss during the 200 epochs was selected as the optimal model, and the generated images, as well as the performance on the test set at this epoch, were thoroughly examined.

## Results

Baseline demographic and clinical characteristics of the included 1640 glaucoma patients are summarized in Table 1. The mean age of the study population was 60.49 ± 15.74 years, with a wide age distribution spanning multiple decades, indicating inclusion of a diverse adult glaucoma cohort. Male patients accounted for 45.8% (751/1640) of the participants.

**Table 1:** Baseline Characteristics of Included 1640 Glaucoma Patients

Characteristics	Values
Diagnosis (%)	
NTG	965 (58.8%)
POAG	631 (38.5%)
PACG	44 (2.7%)
Mean age (yr)	60.49 ± 15.74
Male gender (%)	751 (45.8%)
MD (dB)	–4.50 ± 5.65
PSD (dB)	4.22 ± 3.70
VFI (%)	89.35 ± 16.50
Glaucoma severity (%)	
G1	1217 eyes (74.2%)
G2	251 eyes (15.3%)
G3	172 eyes (10.5%)

dB = decibels; MD = mean deviation; NTG = normal tension glaucoma; PACG = primary angle-closure glaucoma; POAG = primary open-angle glaucoma; PSD = pattern standard deviation; VFI = visual field index.

G1 mild glaucoma grade (–6.0 < MD < 0.0); G2 moderate glaucoma grade (–12.0 < MD ≤ – 6.0); G3 severe glaucoma grade (MD ≤ –12.0).

Regarding VF parameters, the MD was –4.50 ± 5.65 dB, the PSD was 4.22 ± 3.70 dB, and the visual field index (VFI) was 89.35 ± 16.50%, reflecting an overall cohort enriched with early to moderate functional loss.

Based on the Hodapp–Parrish classification, 1217 eyes (74.2%) were classified as having early-stage glaucoma (G1), 251 eyes (15.3%) as moderate glaucoma (G2), and 172 eyes (10.5%) as severe glaucoma (G3).^10^ This distribution indicates that although early-stage glaucoma constituted the majority of the dataset, a substantial proportion of patients with moderate to advanced disease was also included.

### Changes in Generated Images across Epochs

**Figure 2:**
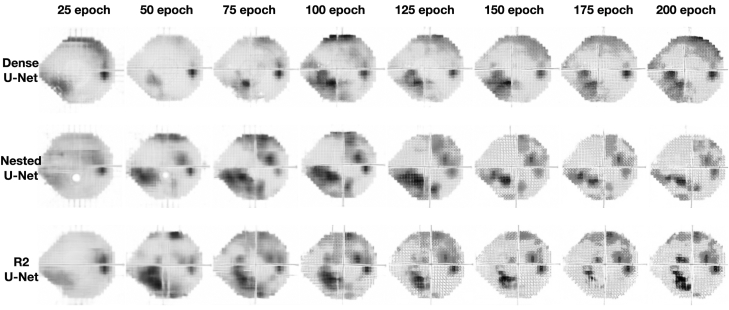
Changes in the predicted images for Dense U-Net, Nested U-Net, and R2 U-Net at every 25 epochs. The images illustrate the improvement in prediction quality over time as training progresses for each architecture.

Figure 2 illustrate the changes in the predicted images for Dense U-Net, Nested U-Net, R2 U-Net at every 25 epochs. It was observed that for all 3 architectures, as training progressed, the predicted images became increasingly closer to the actual VF test results.

### Performance Evolution across Epochs

**Figure 3:**
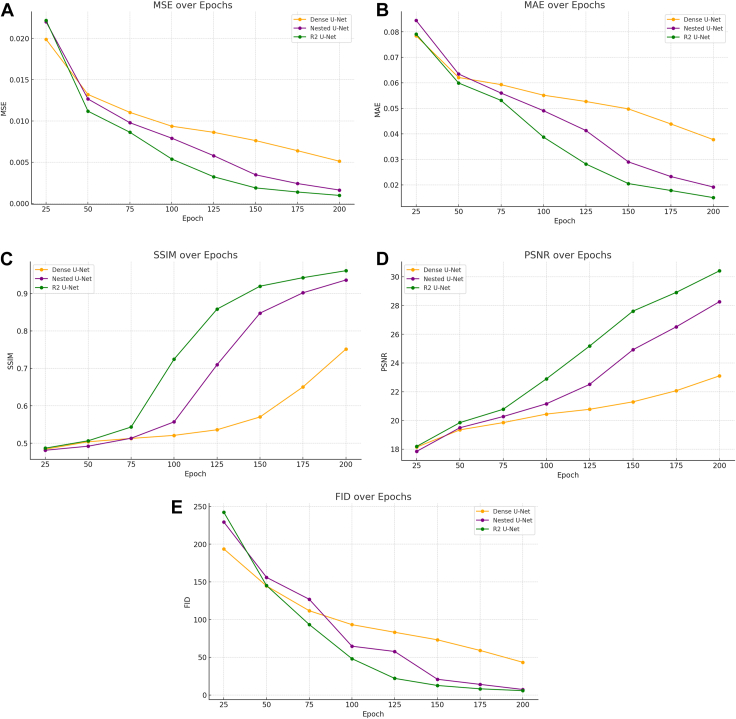
Graph showing the changes in the 5 performance metrics (MSE, MAE, PSNR, SSIM, and FID) on the test set as the number of epochs increases. The graph highlights the learning progression and improvement in model performance with training: **(A)** MSE, **(B)** MAE, **(C)** SSIM, **(D)** PSNR, and **(E)** FID. FID = Fréchet inception distance; MAE = mean absolute error; MSE = mean squared error; PSNR = peak signal-to-noise ratio; SSIM = structural similarity index.

Figure 3 illustrates the changes in the 5 performance metrics on the test set as the number of epochs increased. Dense U-Net, which started with an MSE of around 0.0199 at epoch 25, showed a consistent decrease across the epochs, achieving a final MSE of 0.0051 at epoch 200. Although the improvement was steady, the model lagged behind the others in overall performance. On the other hand, Nested U-Net, which exhibited a slightly higher initial MSE of 0.0220 at epoch 25, significantly improved over time, reaching a final MSE of 0.0016 at epoch 200, thus outperforming Dense U-Net. The model demonstrated substantial improvement after the 100th epoch, where the decrease in MSE became more pronounced. R2 U-Net, which started similarly to the other models with an MSE of 0.0221 at epoch 25, showed the most significant reduction across epochs, ending with the lowest MSE of 0.0009 at epoch 200. It outperformed both Dense U-Net and Nested U-Net in reducing MSE over time, especially after epoch 100. When evaluating MAE, Dense U-Net began with an initial value of 0.0784 at epoch 25 and gradually reduced it to 0.0377 by epoch 200. Although the model showed steady improvement, the final MAE was the highest among the 3 models, indicating larger prediction errors. In contrast, Nested U-Net started with an MAE of 0.0844 at epoch 25 and managed to reduce it significantly to 0.0191 by epoch 200. The model exhibited the fastest improvement in the latter half of the training, with significant reductions occurring after epoch 125. Meanwhile, R2 U-Net, which began with an MAE of 0.0790 at epoch 25, ended with the lowest final MAE of 0.0150 at epoch 200. This model's ability to reduce absolute errors over time was the best among the 3, particularly after epoch 100, where it consistently maintained lower MAE values. In terms of SSIM, Dense U-Net, which started with a value of 0.4839 at epoch 25, gradually improved to 0.7515 by epoch 200. Despite this improvement, the model consistently underperformed compared to the other models, particularly in structural similarity. Nested U-Net, which showed a more rapid increase in SSIM, started at 0.4813 at epoch 25 and reached 0.9359 by epoch 200. This model demonstrated significant improvement, particularly after epoch 100, where the structural similarity improved rapidly. R2 U-Net, which began with an SSIM of 0.4868 at epoch 25, achieved the highest structural similarity, reaching 0.9606 by epoch 200. The model showed consistent improvement throughout the epochs, with particularly strong performance after epoch 150. Regarding PSNR, Dense U-Net, which started with a value of 18.14 at epoch 25, gradually increased to 23.10 by epoch 200. While the model did show improvement, it consistently exhibited the lowest PSNR values, indicating lower image quality. Nested U-Net, which began with a PSNR of 17.84 at epoch 25, improved steadily to a final value of 28.26 at epoch 200, demonstrating better image quality than Dense U-Net, especially after epoch 125, when the PSNR values improved rapidly. R2 U-Net, which started with a PSNR of 18.19 at epoch 25, finished with the highest PSNR of 30.41 at epoch 200, indicating the best overall image quality among the 3 models. Finally, in terms of FID, Dense U-Net, which had an initial FID of 193.71 at epoch 25, managed to reduce it to 43.34 by epoch 200. Although this showed considerable reduction, the model consistently exhibited the highest FID, indicating a larger distribution difference between the predicted and actual images. Nested U-Net, which started with a higher FID of 229.40 at epoch 25, showed rapid improvement, reducing it to 7.28 by epoch 200. The model displayed particularly strong performance in reducing FID after epoch 125. R2 U-Net, which had an initial FID of 242.32 at epoch 25, managed to reduce it to the lowest value of 5.73 by epoch 200. It exhibited the best performance in minimizing distribution differences between predicted and actual images, especially after epoch 150. The final performance metrics of the 3 architectures on the test set at their best-performing epochs are summarized in Table 2.

**Table 2:** Performance on the Test Set at the Best-Performing Epoch for the 3 Architectures

	MSE	MAE	SSIM	PSNR	FID
Dense U-Net	0.0052	0.0371	0.7402	23.0451	40.2544
Nested U-Net	0.0016	0.0187	0.9383	28.3740	8.3055
R2 U-Net	0.0010	0.0152	0.9590	30.2667	5.9889

FID = Fréchet inception distance; MAE = mean absolute error; MSE = mean squared error; PSNR = peak signal-to-noise ratio; SSIM = structural similarity index.

### Performance and Generated Images at the Best-Performing Epoch

**Figure 4:**
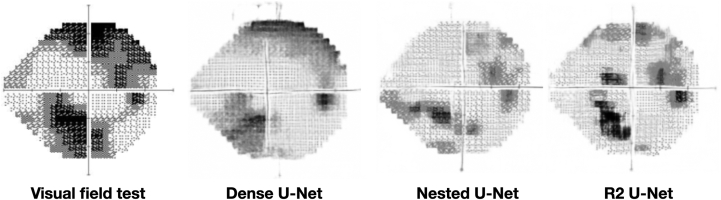
Comparison of the actual visual field test result for the patient from the OCT image in Figure 1, alongside the predicted images from the best-performing models of the 3 architectures used in this study (Dense U-Net, Nested U-Net, and R2 U-Net).

Dense U-Net demonstrated the weakest performance among the 3 models, with the highest MSE (0.005189) and MAE (0.0371), indicating a greater overall prediction error and larger average deviation between the predicted and actual values. The SSIM score of 0.7402 suggests that the structural similarity between the generated and real images was relatively low, and the PSNR of 23.04 confirms that the overall image quality was the poorest. Furthermore, Dense U-Net had the highest FID score (40.25), revealing the largest distribution difference between the predicted and actual images, suggesting a significant gap in image generation accuracy. Nested U-Net performed better than Dense U-Net, with a lower MSE (0.001564) and MAE (0.0187), reflecting more accurate predictions. The SSIM of 0.9383 showed strong structural similarity between the predicted and actual images. Additionally, the PSNR of 28.37 indicated decent image quality, though slightly lower than R2 U-Net. The FID score of 8.31, while still higher than R2 U-Net, demonstrated a moderate distribution difference, marking a solid improvement over Dense U-Net. R2 U-Net exhibited the best performance among the 3 models, with the lowest MSE (0.001017) and MAE (0.0152), indicating a high level of prediction accuracy. The SSIM score of 0.9590 highlighted its ability to produce images that were highly structurally similar to the actual VF images. R2 U-Net also achieved a PSNR of 30.27, demonstrating excellent image quality, and its FID of 5.99 reflected a small distribution difference, making it the most effective model in terms of generating realistic and accurate VF predictions (Table 1). Figure 4 shows the actual VF test result for the patient from the OCT image in Figure 1, alongside the predicted images from the best-performing models of the 3 architectures used in this study.

### Prediction of Visual Field Global Indices Using a Regression-Based Model

We evaluated a regression-based deep learning model and compared its performance with that reported in previous studies (Table 3).^20^^,^^21^ Using a DenseNet121-based convolutional neural network trained on RNFL OCT images, VF global indices, including MD, PSD, and VFI, were predicted.

**Table 3:** Prediction of Visual Field Global Indices Using Regression-Based Deep Learning Models across Studies

Study	Model	*N* (Eyes)	MD *R*^*2*^	MD MAE	PSD *R*^*2*^	PSD MAE	VFI *R*^*2*^	VFI MAE
Christopher et al, 2020	ResNet50	9765	0.70	2.5 dB	0.61	1.5 dB		
Kim et al, 2023	DNN	720	0.62	2.6 dB	0.62	1.8 dB	0.62	6.1%
Present study	DenseNet121	1640	0.54	3.12 dB	0.42	2.16 dB	0.60	8.3%

dB = decibels; DNN = deep neural network; MAE = mean absolute error; MD = mean deviation; PSD = pattern standard deviation; VFI = visual field index.

As summarized in Table 3, the DenseNet121 model achieved an *R*^*2*^ of 0.54 for MD with an MAE of 3.12 dB, an *R*^*2*^ of 0.42 for PSD with an MAE of 2.16 dB, and an *R*^*2*^ of 0.60 for VFI with an MAE of 8.3%. These values were within the range reported in previous studies using ResNet50-based and DNN-based architectures, despite differences in network design and dataset composition.

This regression-based analysis provides an additional quantitative reference for RNFL-based VF global index prediction.

## Discussion

Across all 5 performance metrics, R2 U-Net consistently demonstrated superior results, particularly excelling in MSE, MAE, SSIM, and FID. At the final epoch (200), R2 U-Net achieved the lowest MSE (0.001017) and MAE (0.0152), indicating that its predictions were the most accurate among the models. Its SSIM score of 0.9590 reflected the structural similarity between the predicted VF images and the actual images, while the PSNR value of 30.27 indicated high visual quality. Additionally, the FID value of 5.99 for R2 U-Net showed that the generated image distributions were very close to the real image distributions, making it the best-performing model overall. Nested U-Net also performed well but lagged slightly behind R2 U-Net in several metrics. While it showed strong performance with an MSE of 0.001564 and an MAE of 0.0187, it did not match the precision of R2 U-Net. Its SSIM value of 0.9383 still indicated good structural similarity, and the PSNR of 28.37 pointed to decent image quality, though it was slightly lower than R2 U-Net. The FID of 8.31 suggested that there was more distribution difference between the predicted and actual images than in R2 U-Net. Overall, Nested U-Net performed well, especially during the later stages of training, thanks to its deep architecture and multiple skip connections that helped stabilize performance. In contrast, Dense U-Net exhibited the weakest performance among the models. Despite its initial learning speed, Dense U-Net showed the highest MSE (0.005189) and MAE (0.0371), reflecting greater prediction errors. The SSIM of 0.7402 indicated that its predicted images had the least structural similarity to the actual VF images. Additionally, its PSNR value of 23.04 revealed the poorest image quality, and the FID of 40.25 was the highest, indicating the largest discrepancy between the generated and actual image distributions. These results suggest that while Dense U-Net performed adequately at the start, its overall predictive performance was limited, likely due to the structural limitations of the model.

Although R2 U-Net showed relatively slower initial learning, its recurrent residual connections contributed to improved long-term learning performance, as proposed by Alom et al.^14^ The strong performance of R2 U-Net, particularly in SSIM and PSNR, indicates that this model is highly suitable for predicting VFs from OCT data and can potentially be applied in clinical settings for glaucoma diagnosis and management. Meanwhile, Nested U-Net also showed solid performance, especially during later stages of training, which suggests that models with more complex architectures like UNet++ can significantly improve VF prediction through deep learning. Nested U-Net (UNet++) showed rapid improvement in later stages of learning thanks to its deep network structure and multiple skip connections. This finding supports the work of Zhou et al,^13^ who highlighted the advantages of UNet++ in handling complex image processing tasks. Dense U-Net, although fast in initial learning, struggled to maintain high performance over time. This might be due to the inherent limitations of its architecture, leading to lower-quality predictions compared to the other models. These findings suggest that further model optimization or the use of alternative architectures may help enhance its performance in future research. Dense U-Net, with its densely connected layers maximizing information flow, exhibited strong initial learning speed and final performance, consistent with the work of Huang et al.^11^ This study also confirmed that such dense connections positively impact learning performance.

In interpreting these results, it is noteworthy that the study population demonstrated a wide age distribution, spanning from early adulthood to advanced age, with broad representation across age decades and particularly strong representation between the fourth and eighth decades of life. This age distribution reflects a diverse glaucoma population commonly encountered in routine clinical practice and supports the potential generalizability of the proposed approach across a broad adult age range.

In addition, glaucoma severity in the present cohort was classified using the Hodapp–Parrish criteria, with a substantial proportion of patients presenting with early-stage disease, alongside meaningful representation of moderate and advanced stages.^10^ Despite the relatively higher proportion of early-stage glaucoma—where structural changes may be more subtle—the proposed deep learning framework demonstrated stable and meaningful predictive performance across the overall cohort. These findings suggest that the model is capable of learning clinically relevant structure–function relationships even in earlier stages of glaucoma, highlighting its potential utility in routine clinical settings where early disease detection and functional assessment are particularly important.

This study is significant as it is among the first to apply generative deep learning models, specifically R2 U-Net, Dense U-Net, and Nested U-Net, for predicting VF loss based on RNFL OCT thickness maps. Prior studies have primarily focused on detecting glaucoma or assessing its progression, whereas this study directly generated VF images, offering a new approach to glaucoma diagnosis and management.

Several previous studies have investigated AI-based approaches for glaucoma assessment and VF prediction; however, their objectives and methodological frameworks differ from those of the present study. Most prior work has relied on OCT-derived features for classification tasks or on regression-based prediction of numerical VF indices or partial maps, sometimes incorporating biomechanical information to improve performance.

In contrast, the present study offers several distinct strengths compared with previous AI-based approaches for VF prediction. First, rather than limiting predictions to categorical labels or scalar indices, this study adopts a generative image-to-image learning paradigm using U-Net–based architectures to directly synthesize complete VF pattern images from RNFL OCT data, enabling reconstruction of spatially resolved functional information. Second, the proposed framework relies exclusively on standard RNFL OCT images acquired during routine clinical examinations, without requiring additional biomechanical testing or specialized imaging protocols, thereby enhancing clinical accessibility and practical applicability. Third, this study provides a comprehensive comparison of 3 state-of-the-art generative architectures—R2 U-Net, Dense U-Net, and Nested U-Net—specifically optimized for medical image-to-image translation tasks, elucidating the impact of architectural design on VF image prediction performance. Finally, to further strengthen clinical relevance, we conducted a complementary regression-based analysis using a DenseNet121 architecture to directly predict clinically meaningful VF global indices, including MD, PSD, and VFI. The regression results demonstrated performance comparable to previously reported AI-based studies, supporting the robustness of RNFL-based functional prediction across different modeling paradigms.

To complement the generative modeling results and provide an alternative deep learning perspective on structure–function prediction, we explored a fundamentally different deep learning approach using a DenseNet121-based convolutional neural network. Unlike the U-Net encoder–decoder architecture employed for image-to-image VF generation, DenseNet121 utilizes dense connectivity patterns for feature extraction and is designed for classification and regression tasks. Using this model, we directly predicted VF global indices, including MD, PSD, and VFI, from RNFL OCT images.

As summarized in Table 3, the DenseNet121-based regression model demonstrated competitive performance compared with previously published studies, particularly for VFI prediction (*R*^*2*^ = 0.60). This complementary analysis supports the robustness of RNFL-based functional prediction across different modeling paradigms and provides further insight into the structure–function relationship in glaucoma. While the primary focus of the present study was generative image-to-image VF prediction, these findings suggest that incorporating diverse architectural approaches may further enhance predictive performance and clinical interpretability in future studies.

Despite the success of this study, there are several limitations to note. The study relied solely on RNFL OCT thickness maps, which may not fully capture the complexity of VF loss in glaucoma. Functional impairment in glaucoma is known to be influenced by multiple structural parameters beyond RNFL thickness, including macular ganglion cell complex characteristics and optic nerve head morphological features. Although this single-input approach provides a clear and clinically relevant baseline—given that RNFL OCT is the most commonly acquired structural parameter in routine practice—it nevertheless represents a limitation. Accordingly, this should be viewed as an opportunity for future research to extend the current framework toward multimodal models that integrate additional structural inputs, such as macular ganglion cell complex thickness maps and optic nerve head parameters (e.g., cup-to-disc ratio and rim area), as well as complementary clinical variables. Additional clinical variables such as IOP, medical history, treatment history, axial length, myopia, age, ethnicity, and genetic factors should be incorporated in future studies to enhance predictive accuracy. Moreover, the performance metrics (MSE, MAE, PSNR, SSIM, and FID) used in this study focus on objective, pixel-level differences, which may not entirely reflect human subjective perception of image quality. While SSIM was specifically designed to better align with human visual perception by incorporating structural information, luminance, and contrast, discrepancies between quantitative scores and visual impression may still exist. To address this, future research should consider subjective evaluation methods like mean opinion score or the use of perceptual loss functions to better align model performance with human visual perception. In addition, due to computational resource constraints, the number of epochs was capped at 200. Future studies may explore longer training periods to fully maximize the models' learning potential. Eyes with nonglaucomatous VF defects were also excluded from the present study, which may limit applicability to broader clinical populations. Finally, this study was conducted using data from a single institution, and external validation using independent multicenter datasets was not performed. Although rigorous internal validation was applied, the lack of external validation may limit the generalizability of the findings. Future multicenter studies will be necessary to further validate the robustness and clinical applicability of the proposed model.

Overall, R2 U-Net emerged as the best-performing model, particularly in its ability to generate accurate, high-quality VF images that were structurally similar to the actual VFs. Nested U-Net also performed well, especially during later stages of training, while Dense U-Net showed room for improvement in terms of prediction accuracy and image quality.
